# Statistics of the Sum of Double Random Variables and Their Applications in Performance Analysis and Optimization of Simultaneously Transmitting and Reflecting Reconfigurable Intelligent Surface-Assisted Non-Orthogonal Multi-Access Systems

**DOI:** 10.3390/s24186148

**Published:** 2024-09-23

**Authors:** Bui Vu Minh, Phuong T. Tran, Thu-Ha Thi Pham, Anh-Tu Le, Si-Phu Le, Pavol Partila

**Affiliations:** 1 Faculty of Engineering and Technology, Nguyen Tat Thanh University, Ho Chi Minh City 754000, Vietnam; bvminh@ntt.edu.vn; 2 Wireless Communications Research Group, Faculty of Electrical & Electronics Engineering, Ton Duc Thang University, Ho Chi Minh City 70000, Vietnam; 3 Faculty of Electrical & Electronics Engineering, Ton Duc Thang University, Ho Chi Minh City 70000, Vietnam; 42101299@student.tdtu.edu.vn; 4 Faculty of Electrical Engineering and Computer Science, VSB-Technical University of Ostrava, 17. Listopadu 2172/15, 708 00 Ostrava, Czech Republic; tu.le.anh.st@vsb.cz (A.-T.L.); phu.le.si.st@vsb.cz (S.-P.L.); pavol.partila@vsb.cz (P.P.)

**Keywords:** non-orthogonal multiple access (NOMA), reconfigurable intelligent surface (RIS), simultaneously transmitting and reflecting (STAR), energy harvesting, outage probability (OP), ergodic capacity, symbol error rate

## Abstract

For the future of sixth-generation (6G) wireless communication, simultaneously transmitting and reflecting reconfigurable intelligent surface (STAR-RIS) technology is emerging as a promising solution to achieve lower power transmission and flawless coverage. To facilitate the performance analysis of RIS-assisted networks, the statistics of the sum of double random variables, i.e., the sum of the products of two random variables of the same distribution type, become vitally necessary. This paper applies the statistics of the sum of double random variables in the performance analysis of an integrated power beacon (PB) energy-harvesting (EH)-based NOMA-assisted STAR-RIS network to improve its outage probability (OP), ergodic rate, and average symbol error rate. Furthermore, the impact of imperfect successive interference cancellation (ipSIC) on system performance is also analyzed. The analysis provides the closed-form expressions of the OP and ergodic rate derived for both imperfect and perfect SIC (pSIC) cases. All analyses are supported by extensive simulation results, which help recommend optimized system parameters, including the time-switching factor, the number of reflecting elements, and the power allocation coefficients, to minimize the OP. Finally, the results demonstrate the superiority of the proposed framework compared to conventional NOMA and OMA systems.

## 1. Introduction

Fifth-generation and sixth-generation wireless networks must meet demanding specifications, including high spectral efficiency, huge connectivity, and low latency [1]. Non-orthogonal multiple access (NOMA) has emerged as a promising multiple-access candidate [2,3]. The ability of NOMA to support numerous users via a single resource block effectively increases spectral efficiency. This contrasts with traditional orthogonal multiple access (OMA), in which different users are allocated to different resource blocks (time, frequency, and code). In power-domain (PD) NOMA, users are given varying power levels, and the base station (BS) transmits a single message that combines the signals of all users. Successive interference cancellation (SIC) is used at the receiver side to recover the broadcast symbol and eliminate inter-user interference [4]. The authors of [5] provided a controllable analysis framework for assessing the security and dependability of underlay cognitive radio networks (CRs) that use non-orthogonal multiple access (NOMA), in which a secondary BS broadcasts private data to several uniformly distributed secondary users while an external eavesdropper is present nearby. In [6], the authors examined an Internet-of-Things (IoT) network with two-way relaying non-orthogonal multiple access enabled, where two NOMA users connect via an IoT access point relay using the decode-and-forward (DF) protocol. For an IoT network configuration operating in real time, a deep learning solution with low computational complexity and accurate outage probability (OP) prediction has been established. The performance of the energy-harvesting NOMA system’s uplink and downlink was reviewed by the authors of [7], where they also investigated and tested the closed-form expressions for the OP in a group of two users. The authors of [8] presented a multi-carrier-based method that improves reliability and sum-rate performance by combining transmit diversity with the NOMA protocol. In [9], the authors analyzed the outage performance and optimized simultaneous wireless information and power transfer (SWIPT) in an energy-harvesting wireless sensor network deploying NOMA. The authors of [10] investigated uplink and downlink transmissions in NOMA networks aided by unmanned aerial vehicles. Numerous other papers have also focused on analyzing the OP in cooperative relaying systems employing NOMA [11,12,13,14]. While NOMA has many benefits for communication systems, it cannot address the problems caused by the randomness of wireless channels, which severely limits the performance improvements that NOMA can achieve.

Additionally, the reconfigurable intelligent surface (RIS), a viable option for next-generation wireless communication networks, has also garnered attention for its ability to alter the wireless propagation environment desirably [15,16,17,18]. An RIS is made up of a large number of low-cost passive elements that, by varying their individual phase shifts and amplitudes, reconfigure the propagation of incident wireless signals. By simultaneously adjusting the RIS passive beamforming vector, the transfer time scheduling, and the power splitting ratio under the energy harvest-then-transmit policy protocol, the authors of [19] maximized the sum throughput for an RIS-assisted IoT network. Reconstructing the wireless environment using RIS technology is a unique approach to enhancing NOMA system performance, which is why it is highly advisable to apply RIS technology to the NOMA system [20]. In the context of an RIS-assisted NOMA network combined with the IoT, the authors of [21] explored physical layer security and addressed the issue of non-conclusive eavesdroppers. Two distinct phase shift designs have been investigated to improve the performance of RIS-assisted NOMA systems [22]. In addition, a unique method based on reflection amplitude and phase shift was proposed to find the maximum total rate of all users, including both ideal and non-ideal conditions [23]. By using RISs to produce coordinated multipoint broadcasts, [24] examined how successfully NOMA cellular networks exploit the spectrum. The energy efficiency, OP, and coverage probability of an RIS-assisted two-user NOMA network were examined in [25,26]. In [27], the authors proposed an RIS–NOMA architecture to maximize user service in each orthogonal spatial direction while accounting for hardware constraints. Furthermore, the effects of incorrect successive interference cancellation on the ergodic rate and OP were examined in [28]. The study suggests looking into an RIS-assisted cooperative NOMA network as a means of bridging the gap between RIS-assisted NOMA and user-relaying cooperation.

With the ongoing progress of the technological era, simultaneously transmitting and reflecting reconfigurable intelligent surface (STAR-RIS) technology has emerged as superior to conventional RIS due to its simultaneous transmission and reflection capabilities, enhanced flexibility, and improved energy efficiency [29]. Specifically, STAR-RIS represents a significant advancement over conventional RIS in terms of wireless signal manipulation capabilities. While traditional RIS can only reflect signals, STAR-RIS can both reflect and transmit signals simultaneously. This dual functionality enables STAR-RIS to achieve wider coverage, enhanced signal quality, and reduced interference compared to its conventional counterpart. The enhanced flexibility of STAR-RIS is evident in its ability to independently adjust reflection and transmission coefficients for each element, allowing for precise control over signal direction and intensity. This adaptability enables STAR-RIS to effectively optimize system performance in diverse environments. Moreover, STAR-RIS can leverage both reflected and transmitted signals to enhance signal strength, leading to improved energy efficiency. Numerous papers have demonstrated that STAR-RIS can outperform conventional RIS in various scenarios, including multi-user communication, physical layer security, and wireless power transfer [29,30,31,32,33].

A variety of works integrating STAR-RIS and NOMA in wireless networks have recently been described [31,33,34,35,36,37,38,39,40,41,42,43,44]. While NOMA is designed for massive user access, it experiences problems with cell-edge users due to additional intra-NOMA cluster interference and non-line-of-sight links from the source [45]. This problem can be solved by RIS, especially STAR-RIS. By adding STAR-RISs to NOMA networks, the authors of [31] showed how to greatly increase coverage. STAR-RIS technology was utilized by the authors of [34] to simultaneously improve the required signals and remove inter-cell interference in NOMA-enhanced coordinated multi-point transmission networks. For the STAR-RIS-assisted multiple-input multiple-output system, the weighted sum rate, the sum secrecy rate, and the attainable sum rate of the STAR-RIS–NOMA system were all optimized by the authors of [35,36,37]. To increase the coverage of heterogeneous networks and effectively prevent mutual interference between users, STAR-RIS was used in [33] to modify the decoding order of users. The authors of [38,39] presented several estimated mathematical channel models to examine the OP performance of STAR-RIS–NOMA and STAR-intelligent omni-surfaces-based NOMA multi-cell networks. A realistic transmission and reflection-coupled phase-shift model for STAR-RISs was considered by the authors of [40]. A STAR-RIS partitioning technique for a STAR-RIS–NOMA network was suggested by the authors of [41]. This technique aims to assign the appropriate amount of STAR-RIS components to each user in order to optimize the sum rate and ensure the quality-of-service criteria. For uplink STAR-RIS–NOMA networks, optimization challenges in minimizing power consumption and maximizing OP secrecy were examined in [42,43]. To optimize the sum rate, the authors of [44] examined the resource allocation mechanism in STAR-RIS-assisted multi-carrier OMA and NOMA networks. Table 1 shows the comparison of our work with related work.

In all the above-mentioned studies, the authors did not consider energy-harvesting technology from power beacons. Energy harvesting (EH) from power beacons (PBs) is an emerging technology that enables wireless devices to scavenge energy from ambient radio frequency (RF) signals [46,47,48,49]. This technology has the potential to prolong the lifetime of battery-powered devices and even enable battery-less operation in some cases. Additionally, EH from PBs is an environmentally friendly solution, as it reduces the need for battery disposal and promotes the use of renewable energy sources. Furthermore, power beacons (PBs) have been proposed as a means to provide wireless power transfer to energy-constrained devices in NOMA systems [6,50,51,52]. By integrating PBs with STAR-RIS, it is possible to achieve both wireless information and power transfer, enabling self-sustainable and energy-efficient NOMA networks. For this reason, in this paper, we propose a novel energy-harvesting (EH) framework that integrates PBs, STAR-RISs, and NOMA to achieve enhanced performance in terms of the OP and ergodic rate. We consider both ipSIC and perfect SIC (pSIC) scenarios and derive closed-form expressions for the OP and ergodic rate in both cases. Our analysis reveals the impact of ipSIC on system performance and provides insights into the design and optimization of EH–PB–STAR-RIS–NOMA systems.

One may be worried about the feasibility of this combination of multiple complicated techniques in a single system. But, in general, there are no significant problems in implementing this integrated system in practice. First, the combination of NOMA and STAR-RIS is natural due to their characteristics, and their integration has been confirmed to be feasible and more effective than the conventional NOMA system [45,53]. Second, each contributed technique is primarily processed on independent devices; for example, NOMA operates at the user level, the channel gains of STAR-RIS elements can be controlled at the STAR-RIS, and EH occurs at the source node.

The following is a summary of this manuscript’s main contributions in light of the previously indicated explanations:

- We provide a comprehensive review of the statistics (including the probability density function (PDF) and/or the cumulative distribution function (CDF)) of the sum of double random variables, which plays an important role in the derivation of the performance factors of RIS-assisted networks.
- We propose a novel STAR-RIS-assisted NOMA framework that integrates power beacons (PBs) for energy harvesting, enabling self-sustainable and energy-efficient communication. The probability density function (PDF) and cumulative distribution function (CDF) are considered to validate the accuracy of the approximation method used to model the channel characteristics.
- We derive closed-form expressions for the OP, ergodic rate, and average symbol error rate of the proposed system, considering both perfect and imperfect SIC scenarios. In order to shed light on the design and optimization of EH–PB–STAR-RIS–NOMA systems, we examine the effects of SIC on system performance.
- We provide a comprehensive performance analysis and optimization framework for STAR-RIS-assisted NOMA systems, paving the way for their practical implementation in future wireless networks. Furthermore, analysis and simulation results evaluate the impact of important parameters on performance. To confirm the correctness of our analytical model and to show the advantages of the suggested framework over traditional NOMA and OMA systems, we verify our analytical results through Monte Carlo simulations.

**Table 1:** Comparison of the proposed scheme with other works.

	Our Scheme	[54]	[55]	[56]	[57]
NOMA	✔	X	X	✔	X
STAR-RIS	✔	X	X	X	X
Power Beacon	✔	✔	X	X	✔
Energy Harvesting	✔	✔	✔	✔	✔
Generalized Channels	✔	X	X	X	X
Outage Probability	✔	X	✔	X	✔
Ergodic Capacity	✔	X	X	X	✔
Average SER	✔	X	X	X	X
Throughput	X	✔	X	✔	✔
Optimization	✔	X	X	X	X

## 2. Review of the Statistics of the Sum of Double Random Variables

In this section, we present a review of the statistics of the sum of double random variables (RVs). The exact or approximated forms of the PDF and/or CDF of the sum of double random variables are derived for different distribution types.

The problem of finding the PDF or CDF of double random variables has been a popular topic in wireless communications [58]. A double random variable has the form of Z=XY, where *X* and *Y* follow the same distribution type but may have different distribution parameters. Recently, with the invention of RIS, the interest has extended to the sum of double random variables [59,60], i.e., the sum

(1) $$U=\sum_{i=1}^{n}X_{i}Y_{i},$$

where $X_{i}$ and $Y_{i}$ follow the same distribution but may have different parameters. Each term of this sum corresponds to the end-to-end channel gain from the source to the destination through a specific reflecting element on the RIS. As a result, *U* corresponds to the total received signal at the receiver.

### 2.1. Sum of Double Rayleigh Random Variables

First, let us denote two RVs, *X* and *Y*, and define $Z=\sum_{n=1}^{N}Z_{n}=\sum_{n=1}^{N}X_{n}Y_{n}$. Next, we present the PDF and CDF of *Z* for various RVs. For the Rayleigh RV, the PDF and CDF of $T=\{X_{n},Y_{n}\}$ are, respectively, given as

(2) $$\begin{array}{cc} f_{T}\left(x\right) & =2xe^{-x^{2}}, \end{array}$$

(3) $$\begin{array}{cc} F_{T}\left(x\right) & =1-e^{-x^{2}}, \end{array}$$

Furthermore, the *k*-th moment of *T* is obtained as

(4) $$\begin{array}{c} {\bar{\mu }}_{T}\left(k\right)=E\left[T^{k}\right]=\Gamma \left(\frac{k}{2}+1\right). \end{array}$$

Next, the *k*-th moment of $Z_{n}$ is obtained as

(5) $$\begin{array}{c} {\bar{\mu }}_{Z_{n}}\left(k\right)={\bar{\mu }}_{X_{n}}\left(k\right){\bar{\mu }}_{Y_{n}}\left(k\right). \end{array}$$

Making use of a multinomial expansion [61], the *k*-th moment of $\sum_{n=1}^{N}Z_{n}$ can be obtained as

(6) μ¯Z(k)=∑k1=0k∑k2=0k1…∑kQ−1=0kQ−2kk1k1k2…kQ−2Q−1×μ¯Zn(k−k1)μ¯Zn(k1−k2)…μ¯Zn(kQ−1),

Next, we can fit *Z* as the Gamma distribution, with the shape parameter ξ and the inverse scale parameter ζ, which are given by

(7a) $$\begin{array}{cc} \; & \xi =\frac{{\left(E\left\{Z\right\}\right)}^{2}}{\mathrm{Var}\left\{Z\right\}}=\frac{{\left[{\bar{\mu }}_{Z}\left(2\right)\right]}^{2}}{{\bar{\mu }}_{Z}\left(4\right)-{\left[{\bar{\mu }}_{Z}\left(2\right)\right]}^{2}}, \end{array}$$

(7b) $$\begin{array}{cc} \; & \zeta =\frac{E\left\{Z\right\}}{\mathrm{Var}\left\{Z\right\}}=\frac{{\bar{\mu }}_{Z}\left(2\right)}{{\bar{\mu }}_{Z}\left(4\right)-{\left[{\bar{\mu }}_{Z}\left(2\right)\right]}^{2}}. \end{array}$$

Furthermore, the corresponding PDF and CDF of $A_{i}$ are given by

(8) $$\begin{array}{cc} f_{Z}\left(x\right) & =\frac{x^{\xi -1}\zeta_{i}^{\xi }e^{-x\zeta_{i}}}{\Gamma (\xi )}, \end{array}$$

(9) $$\begin{array}{cc} F_{Z}\left(x\right) & =\frac{\gamma (\xi ,\zeta x)}{\Gamma (\xi )}=1-\frac{\Gamma (\xi ,\zeta x)}{\Gamma (\xi )}, \end{array}$$

where Γ(.) is the Gamma function, and γ(.,.) and Γ(.,.) are the lower and upper incomplete Gamma functions, respectively.

### 2.2. Sum of Double Nakagami-*m* Random Variables

In this subsection, the PDF and CDF of *T* can be represented as

(10) $$\begin{array}{cc} f_{T}\left(x\right) & =\frac{2m_{T}}{\Gamma (m_{T})}x^{2m_{T}-1}e^{-m_{T}x^{2}}, \end{array}$$

(11) $$\begin{array}{cc} F_{T}\left(x\right) & =\frac{1}{\Gamma (m_{T})}\gamma m_{T},m_{T}x^{2}, \end{array}$$

where $m_{T}$ is the shape parameter and Γ(.) is the Gamma distribution [62]. Furthermore, the *k*-th moment of *T* is obtained as   

(12) $$\begin{array}{c} {\bar{\mu }}_{T}\left(k\right)=\frac{\Gamma (m_{T}+k/2)}{\Gamma \left(m_{T}\right){\sqrt{m_{T}}}^{k}}. \end{array}$$

Next, the *k*-th moment of $Z_{n}$ and *Z* in this case can be obtained similarly to those in (5) and (6).

Similar to the Rayleigh RV, the corresponding PDF and CDF of *Z* are, respectively, given by

(13) $$\begin{array}{cc} f_{Z}\left(x\right) & =\frac{x^{\xi -1}\zeta_{i}^{\xi }e^{-x\zeta_{i}}}{\Gamma (\xi )}, \end{array}$$

(14) $$\begin{array}{cc} F_{Z}\left(x\right) & =\frac{\gamma (\xi ,\zeta x)}{\Gamma (\xi )}=1-\frac{\Gamma (\xi ,\zeta x)}{\Gamma (\xi )}. \end{array}$$

where ξ and ζ defined given similarly to (7a) and (7b).

### 2.3. Sum of Double κ-μ Random Variables

We can express the PDF and CDF of *T* as [63]

(15) $$\begin{array}{cc} f_{T}\left(x\right)=\frac{\mu {\left(1+\kappa \right)}^{\frac{\mu +1}{2}}}{\kappa^{\frac{\mu -1}{2}}e^{\mu \kappa }}x^{\frac{\mu -1}{2}}e^{-\mu \left(1+\kappa \right)x}I_{\mu -1}\left(2\mu \sqrt{\kappa \left(1+\kappa \right)x}\right), & x>0, \end{array}$$

and

(16) $$F_{T}\left(z\right)=1-Q_{\mu }\left(\sqrt{2\kappa \mu },\sqrt{2\left(1+\kappa \right)}x\right),$$

where Qca,b=a1−c∫b∞xce−x2+a2x2+a222Ic−1axdx specifies the generalized Marcum Q-function and $I_{v}\left(.\right)$ is the modified Bessel function of the first kind and order *v*. Furthermore, the *k*-th moment of *T* is obtained as [63]

(17) μ¯T(k)=Γ(μ+k/2)exp(−κμ)Γ(μ)[(1+κ)μ]k/2F11(μ+k/2;μ;κμ),

Next, the *k*-th moment of $Z_{n}$ and *Z* in this case can be obtained similarly to those in (5) and (6). Similar to the Rayleigh RV, the corresponding PDF and CDF of *Z* are, respectively, given by

(18) $$\begin{array}{cc} f_{Z}\left(x\right) & =\frac{x^{\xi -1}\zeta_{i}^{\xi }e^{-x\zeta_{i}}}{\Gamma (\xi )}, \end{array}$$

(19) $$\begin{array}{cc} F_{Z}\left(x\right) & =\frac{\gamma (\xi ,\zeta x)}{\Gamma (\xi )}=1-\frac{\Gamma (\xi ,\zeta x)}{\Gamma (\xi )}. \end{array}$$

where ξ and ζ are defined similarly to (7a) and (7b).

## 3. System Model and Channel Characteristics

### 3.1. System Model

**Figure 1:**
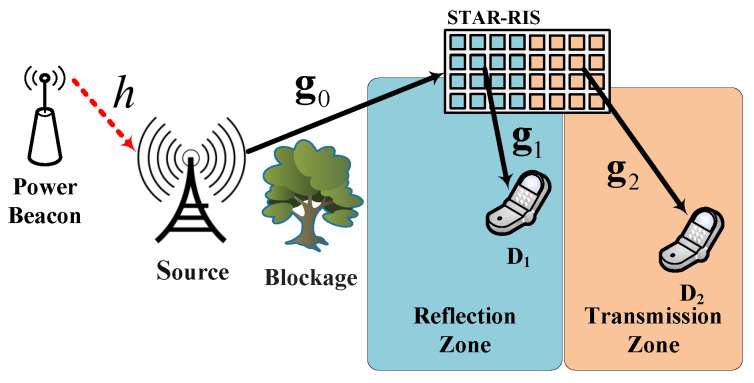
STAR-RIS-assisted NOMA system.

We consider a wireless communication system, as depicted in Figure 1, where a signal is transmitted from the source (S) to the destination ($D_{i}$ with i∈1,2). The source’s transmitted signal is reflected by the STAR-RIS, which is made up of 2Q elements after the power beacon (PB) sends wireless energy to S. Furthermore, we adopt the switching mode used in [64], i.e., 2Q elements are divided into two equal parts to serve $D_{1}$ and $D_{2}$. Furthermore, using the received RF signal from the PB node, we assume that source S harvests energy for ατ seconds, where τ is the frame period and 0<α<1. The energy captured by S is utilized to send data to the destination for the remainder of the frame, i.e., $\left(1-\alpha \right)\tau$ seconds. We calculate the throughput for various IRS setups when used as a reflector. We also recommend optimizing the harvesting period to enhance throughput. A short harvesting period results in little gathered energy and poor throughput. If the harvesting length is sufficiently long, there may not be enough time for data transmission during the remainder of the frame, i.e., $\left(1-\alpha \right)\tau$.

S gathers energy from the RF signal received from the PB. The amount of energy collected is equal to

(20) $$E=\eta \alpha \tau P_{PB}{\left|\bar{h}\right|}^{2},$$

where α is the fraction of the block time in which S harvests energy from the PB’s information signal,0<η<1 denotes the energy conversion efficiency, $P_{PB}$ is the power of the PB node, *h* is the channel coefficient between the PB and S, and $\bar{h}\overset{\Delta }{\;=}he^{j\theta^{h}}$ denotes the channel from the PB to S, where $\theta^{h}$ represents the channel phases that are uniformly distributed between 0 and 2π. The average power of the channel coefficient *h* is $E\left\{{\left|h\right|}^{2}\right\}=\lambda_{h}$, where $E\left\{X\right\}$ is the expectation operation of *X*.

As in several prior works, we assume that all the harvested energy is used during the information transmission phase. Hence, the transmit power of the source is given by

(21) $$P_{S}=\frac{E}{\left(1-\alpha \right)\tau }=\frac{\eta \alpha P_{PB}}{\left(1-\alpha \right)}{\left|\bar{h}\right|}^{2}.$$

S uses the gathered energy to transmit the separate signal $D_{i}$ via the STAR-RIS paradigm during the wireless information transfer (WIT) phase. As a result, the signals received at $D_{1}$ and $D_{2}$ are represented as

(22a) $$\begin{array}{cc} \; & y_{1}=g_{1}^{H}\Phi_{1}g_{0}\left(\sqrt{a_{1}P_{S}}x_{1}+\sqrt{a_{2}P_{S}}x_{2}\right)+n_{1}, \end{array}$$

(22b) $$\begin{array}{cc} \; & y_{2}=g_{2}^{H}\Phi_{2}g_{0}\left(\sqrt{a_{1}P_{S}}x_{1}+\sqrt{a_{2}P_{S}}x_{2}\right)+n_{2}, \end{array}$$

where $P_{S}$ denotes the transmit power of *S*, and $x_{i}$, $i\in \left\{1,2\right\}$ is the normalized transmit signal with unit energy such that $E\left\{{\left|x_{i}\right|}^{2}\right\}=1$. We have $a_{1}$ and $a_{2}$, which are the power allocation coefficients satisfying $a_{2}>a_{1}$ and $a_{2}+a_{1}=1$. We define $g_{0}=\left[{\bar{g}}_{0,1},\ldots ,{\bar{g}}_{0,q},\ldots ,{\bar{g}}_{0,Q}\right]$, $g_{1}={\left[{\bar{g}}_{1,1},\ldots ,{\bar{g}}_{1,n},\ldots ,{\bar{g}}_{1,Q}\right]}^{H}$, and $g_{2}={\left[{\bar{g}}_{2,1},\ldots ,{\bar{g}}_{2,n},\ldots ,{\bar{g}}_{2,Q}\right]}^{H}$, where ${\bar{g}}_{0,q}=g_{0,q}e^{j\theta_{q}^{g_{0}}}$, ${\bar{g}}_{1,q}=g_{1,q}e^{j\theta_{q}^{g_{1}}}$, and ${\bar{g}}_{2,q}=g_{2,q}e^{j\theta_{q}^{g_{2}}}$ denote the channel coefficients from S to the STAR-RIS, from the STAR-RIS to $D_{1}$ and from the STAR-RIS to $D_{2}$, respectively. Meanwhile, the phases of the channels, i.e., $\theta_{q}^{g_{0}}$, $\theta_{q}^{g_{1}}$, and $\theta_{q}^{g_{2}}$ are uniformly distributed from 0 to 2π.

Let $\Phi_{1}=diag\left(\sqrt{\beta_{1}^{g_{1}}}e^{j\phi_{1}^{g_{1}}},\ldots ,\sqrt{\beta_{q}^{g_{1}}}e^{j\phi_{q}^{g_{1}}},\ldots ,\sqrt{\beta_{Q}^{g_{1}}}e^{j\phi_{Q}^{g_{1}}}\right)$ and $\Phi_{2}=diag\left(\sqrt{\beta_{1}^{g_{2}}}e^{j\phi_{1}^{g_{2}}},\ldots ,\sqrt{\beta_{q}^{g_{2}}}e^{j\phi_{q}^{g_{2}}},\ldots ,\sqrt{\beta_{Q}^{g_{2}}}e^{j\phi_{Q}^{g_{2}}}\right)$ denote the reflection and transmission coefficient matrices of the STAR-RIS, where $\beta_{q}^{g_{1}}$, $\beta_{q}^{g_{2}}\in \left[0,1\right]$ denote the amplitude of the *q*th STAR-RIS element and $\phi_{q}^{g_{1}}$, $\phi_{q}^{g_{2}}\in \left[0,2\pi \right)$ denote the phase shift of the *q*th STAR-RIS element. The additive Gaussian noise is given by $n_{i}∼CN\left(0,\sigma_{i}^{2}\right)$, which has a mean of 0 and variance of $\sigma_{i}^{2}$.

For the STAR-RIS–NOMA network, $D_{1}$ first decodes $D_{2}$’s signal and then decodes its own signal by eliminating $D_{2}$’s signal with SIC. Assuming perfect channel state information (CSI), the signal-plus-interference-to-noise ratio (SINR) of these two processes is expressed as

(23a) $$\begin{array}{cc} \; & \gamma_{D_{2}\to D_{1}}=\frac{a_{2}P_{S}{\left|g_{1}^{H}\Phi_{1}g_{0}\right|}^{2}}{a_{1}P_{S}{\left|g_{1}^{H}\Phi_{1}g_{0}\right|}^{2}+\sigma_{1}^{2}}, \end{array}$$

(23b) $$\begin{array}{cc} \; & \gamma_{D_{1}}=\left\{\begin{array}{cc} \frac{a_{1}P_{S}{\left|g_{1}^{H}\Phi_{1}g_{0}\right|}^{2}}{\ell a_{2}P_{S}{\left|g_{1}^{H}\Phi_{1}g_{0}\right|}^{2}+\sigma_{1}^{2}} & \;\;\;\;\;\;\;\;\;,0<\ell \leq 1\\ \frac{a_{1}P_{S}{\left|g_{1}^{H}\Phi_{1}g_{0}\right|}^{2}}{\sigma_{1}^{2}} & ,\ell =0 \end{array}\right. \end{array}$$

where *ℓ*, 0≤ℓ≤1, represents the efficiency of SIC for $x_{1}$ at $D_{1}$. The cases ℓ=0 and ℓ=1 correspond to perfect SIC (pSIC) and imperfect SIC (ipSIC), respectively.

Submitting (21) into (23a) and (23b), $\gamma_{D_{2}\to D_{1}}$ and $\gamma_{D_{1}}$ are given by

(24a) $$\begin{array}{cc} \; & \gamma_{D_{2}\to D_{1}}=\frac{a_{2}\delta P_{PB}{\left|\bar{h}g_{1}^{H}\Phi_{1}g_{0}\right|}^{2}}{a_{1}\delta P_{PB}{\left|hg_{1}^{H}\Phi_{1}g_{0}\right|}^{2}+\sigma_{1}^{2}}, \end{array}$$

(24b) $$\begin{array}{cc} \; & \gamma_{D_{1}}=\left\{\begin{array}{cc} \frac{a_{1}\delta P_{PB}{\left|\bar{h}g_{1}^{H}\Phi_{1}g_{0}\right|}^{2}}{\ell a_{2}\delta P_{PB}{\left|\bar{h}g_{1}^{H}\Phi_{1}g_{0}\right|}^{2}+\sigma_{1}^{2}} & ,0<\ell \leq 1\\ \frac{a_{1}\delta P_{PB}{\left|\bar{h}g_{1}^{H}\Phi_{1}g_{0}\right|}^{2}}{\sigma_{1}^{2}} & ,\ell =0 \end{array}\right. \end{array}$$

where δ=ηαρηαρ1−α1−α.

It should be noted that we can rewrite (24a) and (24b) as

(25a) $$\begin{array}{cc} \; & \gamma_{D_{2}\to D_{1}}=\frac{a_{2}\delta \rho {\left|\bar{h}g_{1}^{H}\Phi_{1}g_{0}\right|}^{2}}{a_{1}\delta \rho {\left|\bar{h}g_{1}^{H}\Phi_{1}g_{0}\right|}^{2}+1}, \end{array}$$

(25b) $$\begin{array}{cc} \; & \gamma_{D_{1}}=\left\{\begin{array}{cc} \frac{a_{1}\delta \rho {\left|\bar{h}g_{1}^{H}\Phi_{1}g_{0}\right|}^{2}}{\ell a_{2}\delta \rho {\left|\bar{h}g_{1}^{H}\Phi_{1}g_{0}\right|}^{2}+\sigma_{1}^{2}} & ,0<\ell \leq 1\\ a_{1}\delta \rho {\left|\bar{h}g_{1}^{H}\Phi_{1}g_{0}\right|}^{2} & ,\ell =0 \end{array}\right. \end{array}$$

where $\rho =\frac{P_{PB}}{\sigma_{1}^{2}}=\frac{P_{PB}}{\sigma_{2}^{2}}$ is the signal-to-noise ratio (SNR) at the PB.

Unlike $D_{1}$, $D_{2}$ directly detects its own signal; thus, its SINR is given as

(26) $$\gamma_{D_{2}}=\frac{a_{2}\delta \rho {\left|\bar{h}g_{2}^{H}\Phi_{2}g_{0}\right|}^{2}}{a_{1}\delta \rho {\left|\bar{h}g_{2}^{H}\Phi_{2}g_{0}\right|}^{2}+1}.$$

We denote the phases of the cascaded channels as $\phi_{1}=\theta^{h}+\theta_{q}^{g_{0}}+\theta_{q}^{g_{1}}$ and $\phi_{2}=\theta^{h}+\theta_{q}^{g_{0}}+\theta_{q}^{g_{2}}$. Furthermore, we assume that $\phi_{q}^{g_{1}}=-\phi_{1}$ and $\phi_{q}^{g_{2}}=-\phi_{2}$, and then the effective cascade channels from S to users are expressed as ${\left|h\sum_{q=1}^{Q}g_{0,q}g_{1,q}\right|}^{2}$ and ${\left|h\sum_{q=1}^{Q}g_{0,q}g_{2,q}\right|}^{2}$, respectively. In this case, Equations (25a), (25b) and (26) are rewritten as   

(27a) $$\begin{array}{cc} \; & \gamma_{D_{2}\to D_{1}}=\frac{a_{2}\delta \rho A_{1}}{a_{1}\delta \rho A_{1}+1}, \end{array}$$

(27b) $$\begin{array}{cc} \; & \gamma_{D_{1}}=\left\{\begin{array}{cc} \frac{a_{1}\delta \rho A_{1}}{\ell a_{2}\delta \rho A_{1}+1} & ,0<\ell \leq 1\\ a_{1}\delta \rho A_{1} & ,\ell =0 \end{array}\right. \end{array}$$

(27c) $$\begin{array}{cc} \; & \gamma_{D_{2}}=\frac{a_{2}\delta \rho A_{2}}{a_{1}\delta \rho A_{2}+1}, \end{array}$$

where $A_{1}={\left|h\sum_{q=1}^{Q}g_{0,q}g_{1,q}\right|}^{2}$ and $A_{2}={\left|h\sum_{q=1}^{Q}g_{0,q}g_{2,q}\right|}^{2}$.

### 3.2. Channel Characteristics

First, we define $B_{i}=\sum_{q=1}^{Q}g_{0,q}g_{i,q}$ and present the distribution for the sum of double RVs in Section 2. Thus, the *k*-th moment of $B_{i}$ is given by

(28) $$\begin{array}{c} {\bar{\mu }}_{B_{i}}\left(k\right)=\frac{\Gamma (\xi +k)}{\gamma \left(\xi \right)\zeta^{k}}, \end{array}$$

Based on the independence of $B_{i}$ and *h*, the *k*-th moment of $A_{i}$ can be obtained as

(29) $$\begin{array}{c} {\bar{\mu }}_{A_{i}}\left(k\right)={\bar{\mu }}_{B_{i}}\left(k\right){\bar{\mu }}_{h}\left(k\right), \end{array}$$

where ${\bar{\mu }}_{h}\left(k\right)$ is presented in Section 2. Next, we can fit $A_{i}$ as the Gamma distribution, with the shape parameter $\xi_{i}$ and the inverse scale parameter $\zeta_{i}$, which are given by

(30a) $$\begin{array}{cc} \; & \xi_{i}=\frac{{\left(E\left\{A_{i}\right\}\right)}^{2}}{\mathrm{Var}\left\{A_{i}\right\}}=\frac{{\left[\mu_{A_{i}}\left(2\right)\right]}^{2}}{\mu_{A_{i}}\left(4\right)-{\left[\mu_{A_{i}}\left(2\right)\right]}^{2}}, \end{array}$$

(30b) $$\begin{array}{cc} \; & \zeta_{i}=\frac{E\left\{A_{i}\right\}}{\mathrm{Var}\left\{A_{i}\right\}}=\frac{\mu_{A_{i}}\left(2\right)}{\mu_{A_{i}}\left(4\right)-{\left[\mu_{A_{i}}\left(2\right)\right]}^{2}}. \end{array}$$

Furthermore, the corresponding PDF and CDF of $A_{i}$ are given by

(31) $$\begin{array}{cc} f_{A_{i}}\left(x\right) & =\frac{x^{\xi_{i}-1}\zeta_{i}^{\xi_{i}}e^{-x\zeta_{i}}}{\Gamma (\xi_{i})}, \end{array}$$

(32) $$\begin{array}{cc} F_{A_{i}}\left(x\right) & =\frac{\gamma (\xi_{i},\zeta_{i}x)}{\Gamma (\xi_{i})}=1-\frac{\Gamma (\xi_{i},\zeta_{i}x)}{\Gamma (\xi_{i})}, \end{array}$$

where Γ(.) is the Gamma function, and γ(.,.) and Γ(.,.) are the lower and upper incomplete Gamma functions, respectively.

## 4. Outage Probability Analysis

The performance of the STAR-RIS-assisted NOMA system is characterized in terms of the OP. The next section discusses the OP caused by the channel reciprocity of $D_{2}$ and $D_{1}$.

### 4.1. Outage Probability $D_{2}$

The following scenario depicts NOMA failure incidents at $D_{2}$. $D_{2}$ can reliably locate $x_{2}$. The OP of $D_{2}$ can therefore be expressed as

(33) $$\begin{array}{cc} P_{D_{2}}= & 1-\mathrm{Pr}\left(\gamma_{D_{2}}>\gamma_{th2}\right)\\ = & 1-\mathrm{Pr}\left(A_{2}>\upsilon_{2}\right)\\ = & F_{A_{2}}\left(\upsilon_{2}\right), \end{array}$$

where $\gamma_{th2}=2^{\frac{R_{2}}{1-\alpha }}-1$, with $R_{2}$ being the target rate at $D_{2}$ to detect $x_{2}$, and $\upsilon_{2}=\frac{\gamma_{th2}}{\delta \rho \left(a_{2}-\gamma_{th2}a_{1}\right)}$.

Substituting (32) into (33), the OP at $D_{2}$ is represented as

(34) $$P_{D_{2}}=1-\frac{\Gamma (\xi_{2},\zeta_{2}\upsilon_{2})}{\Gamma (\xi_{2})}.$$

### 4.2. Outage Probability $D_{1}$

In a STAR-RIS-assisted NOMA system, $D_{1}$ outage events are defined as follows: (i) $D_{1}$ is unable to detect the information $x_{2}$; and (ii) $D_{1}$ is unable to detect $x_{2}$ but can successfully decode $x_{1}$. To simplify the current analysis, the complementary events of $x_{1}$ reflect the OP. As a result, for 0<ℓ≤1, the OP at $D_{1}$ with ipSIC for a STAR-RIS-supported NOMA system is given as

(35) $$\begin{array}{cc} P_{D_{1}}^{ipSIC}= & 1-\mathrm{Pr}\left(\gamma_{D_{2}\to D_{1}}>\gamma_{th2},\gamma_{D_{1}}>\gamma_{th1}\right)\\ = & 1-\mathrm{Pr}\left(A_{1}>\upsilon_{2},A_{1}>\upsilon_{3}\right)\\ = & 1-\mathrm{Pr}\left(A_{1}>\upsilon_{\max}\right)\\ = & F_{A_{1}}\left(\upsilon_{\max}\right), \end{array}$$

where $\gamma_{th1}=2^{\frac{R_{1}}{1-\alpha }}-1$, with $R_{1}$ being the target rate at $D_{1}$ to detect $x_{1}$, $\upsilon_{3}=\frac{\gamma_{th1}}{\delta \rho \left(a_{1}-\gamma_{th1}\ell a_{2}\right)}$, and $\upsilon_{\max}=\max\left(\upsilon_{2},\upsilon_{3}\right)$.

Substituting (32) into (35), the OP at $D_{1}$ with ipSIC is expressed as

(36) $$P_{D_{1}}^{ipSIC}=1-\frac{\Gamma (\xi_{1},\zeta_{1}\upsilon_{\max})}{\Gamma (\xi_{1})}.$$

Similar to (36), for the case ℓ=0, the OP at $D_{1}$ with pSIC is calculated as follows:

(37) $$P_{D_{1}}^{pSIC}=1-\frac{\Gamma (\xi_{1},\zeta_{1}{\tilde{\upsilon }}_{\max})}{\Gamma (\xi_{1})},$$

where ${\tilde{\upsilon }}_{\max}=\max\left(\upsilon_{2},\upsilon_{1}\right)$ and $\upsilon_{3}=\frac{\gamma_{th1}}{\delta \rho a_{1}}$.

### 4.3. Diversity Analysis

To shed light on the performance of the proposed STAR-RIS-assisted NOMA system, the asymptotic OP in the high SNR regime (ρ→∞) is shown. The diversity order is defined as follows:

(38a) $$\begin{array}{cc} \; & \begin{array}{cc} d_{1}^{★}=-\lim_{\rho \to \infty }\frac{\log\left(P_{D_{1}}^{\infty ,★}\left(\rho \right)\right)}{\log\left(\rho \right)} & ,★\in \left\{ipSIC,pSIC\right\} \end{array}, \end{array}$$

(38b) $$\begin{array}{cc} \; & d_{2}=-\lim_{\rho \to \infty }\frac{\log\left(P_{D_{2}}^{\infty }\left(\rho \right)\right)}{\log\left(\rho \right)}, \end{array}$$

where $P_{D_{1}}^{\infty ,★}\left(\rho \right)$ and $P_{D_{2}}^{\infty }\left(\rho \right)$ denote the asymptotic OPs of $D_{1}$ and $D_{2}$, respectively.

When ρ→∞, the asymptotic form of $F_{A_{i}}\left(z\right)$ is as follows [62]:

(39) $$F_{A_{i}}\left(x\right)\approx \frac{1}{\xi_{i}!}{\left(\zeta_{i}x\right)}^{\xi_{i}}.$$

Plugging (37), (36) and (34) into (39), the asymptotic OPs of $P_{D_{1}}^{\infty ,ipSIC}$, $P_{D_{1}}^{\infty ,pSIC}$, and $P_{D_{2}}^{\infty }$ can be expressed as   

(40a) $$\begin{array}{cc} \; & P_{D_{1}}^{\infty ,ipSIC}=\frac{{\left(\zeta_{1}\upsilon_{\max}\right)}^{\xi_{1}}}{\xi_{1}!}, \end{array}$$

(40b) $$\begin{array}{cc} \; & P_{D_{1}}^{\infty ,pSIC}=\frac{{\left(\zeta_{1}{\tilde{\upsilon }}_{\max}\right)}^{\xi_{1}}}{\xi_{1}!}, \end{array}$$

(40c) $$\begin{array}{cc} \; & P_{D_{2}}^{\infty }=\frac{{\left(\zeta_{2}\upsilon_{2}\right)}^{\xi_{2}}}{\xi_{2}!}. \end{array}$$

## 5. Evaluation of Ergodic Rate

Other capacity performance gaps arise because the outage performance of the destination users $D_{1}$ and $D_{2}$ is expected to exhibit a performance gap due to differing power allocation parameters in the NOMA system. As a result, this inspires us to conduct an additional evaluation of the suggested STAR-RIS-assisted NOMA system in terms of the ergodic rate.

### 5.1. Ergodic Rate of $D_{2}$

In this section, we investigate the system’s ergodic rate. The ergodic rate of $D_{2}$ for the NOMA downlink is given by

(41) $$C_{D_{2}}=E\left\{\log_{2}\left(1+\gamma_{D_{2}}\right)\right\}.$$

By the definition of the expectation operator and after integration by parts, $C_{D_{2}}$ can be expressed as

(42) $$C_{D_{2}}=\frac{1}{\ln2}\int_{0}^{\frac{a_{2}}{a_{1}}}\frac{1}{1+x}{\bar{F}}_{A_{2}}\left(\frac{x}{\delta \rho \left(a_{2}-xa_{1}\right)}\right)dx,$$

where ${\bar{F}}_{A_{2}}\left(x\right)$ denotes the complementary CDF of $A_{2}$, i.e., ${\bar{F}}_{A_{2}}\left(x\right)=1-F_{A_{2}}\left(x\right)$. By changing variables to $t\leftarrow \frac{x}{\delta \rho \left(a_{2}-xa_{1}\right)}$ and after a few steps, (42) can be further derived as

(43) $$C_{D_{2}}=\frac{1}{\ln2}\int_{0}^{\infty }\left[\frac{1}{t+\Theta_{1}}-\frac{1}{t+\Theta_{2}}\right]{\bar{F}}_{A_{2}}\left(t\right)dt,$$

where $\Theta_{1}={\left[\delta \rho \left(a_{2}+a_{1}\right)\right]}^{-1}$ and $\Theta_{2}={\left(\delta \rho a_{1}\right)}^{-1}$.

By substituting (34) into (43), $C_{D_{2}}$ can be written as

(44) $$C_{D_{2}}=\frac{1}{\ln2\Gamma (\xi_{2})}\int_{0}^{\infty }\left[\frac{1}{t+\Theta_{1}}-\frac{1}{t+\Theta_{2}}\right]\Gamma \left(\xi_{2},\zeta_{2}t\right)dt.$$

To solve the integrals in (44), we use the following transformations with the Meijer G-function [6]

(45) $$\Gamma \left(\alpha ,x\right)=G_{1,2}^{2,0}\left(x\left|\begin{array}{c} 1\\ \alpha ,0 \end{array}\right.\right).$$

By converting the required functions in (44) into Meijer G-functions using the aforementioned equalities in (45), we can solve the integral as follows:

(46) $$\begin{array}{cc} C_{D_{2}}= & \frac{1}{\ln2\Gamma (\xi_{2})}\int_{0}^{\infty }\left[\frac{1}{t+\Theta_{1}}-\frac{1}{t+\Theta_{2}}\right]G_{1,2}^{2,0}\left(\zeta_{2}t\left|\begin{array}{c} 1\\ \xi_{2},0 \end{array}\right.\right)dt\\ = & \frac{1}{\ln2\Gamma (\xi_{2})}\left[\frac{1}{t+\Theta_{1}}G_{1,2}^{2,0}\left(\zeta_{2}t\left|\begin{array}{c} 1\\ \xi_{2},0 \end{array}\right.\right)dt-\frac{1}{t+\Theta_{2}}G_{1,2}^{2,0}\left(\zeta_{2}t\left|\begin{array}{c} 1\\ \xi_{2},0 \end{array}\right.\right)dt\right]. \end{array}$$

Then, with the aid of [62], Equation (7.811.5), $C_{D_{2}}$ is obtained as

(47) $$C_{D_{2}}=\frac{1}{\ln2\Gamma (\xi_{2})}\left[G_{2,3}^{3,1}\left(\zeta_{2}\Theta_{1}\left|\begin{array}{c} 0,1\\ 0,\xi_{2},0 \end{array}\right.\right)-G_{2,3}^{3,1}\left(\zeta_{2}\Theta_{2}\left|\begin{array}{c} 0,1\\ 0,\xi_{2},0 \end{array}\right.\right)\right].$$

### 5.2. Ergodic Rate of $D_{1}$ with Imperfect SIC

The achievable rate of the considered system at $D_{1}$ with 0<ℓ≤1 is given as

(48) $$\begin{array}{cc} C_{D_{1}}^{ipSIC}= & E\left\{\log_{2}\left(1+\gamma_{D_{1}}\right)\right\}\\ = & E\left\{\log_{2}\left(1+\frac{a_{1}\delta \rho A_{1}}{\ell a_{2}\delta \rho A_{1}+1}\right)\right\}\\ = & \frac{1}{\ln2}\int_{0}^{\frac{a_{1}}{\ell a_{2}}}\frac{1}{1+x}\left[1-F_{A_{1}}\left(\frac{x}{\delta \rho \left(a_{1}-x\ell a_{2}\right)}\right)\right]dx. \end{array}$$

By replacing the variable $t=\frac{x}{\delta \rho \left(a_{1}-x\ell a_{2}\right)}$ in (48) and after a few steps, $C_{D_{1}}^{ipSIC}$ can be further derived as

(49) $$C_{D_{1}}^{ipSIC}=\frac{1}{\ln2}\int_{0}^{\infty }\left[\frac{1}{t+\Theta_{3}}-\frac{1}{t+\Theta_{4}}\right]\left[1-F_{A_{1}}\left(t\right)\right]dt,$$

where $\Theta_{3}={\left[\delta \rho \left(a_{1}+\ell a_{2}\right)\right]}^{-1}$ and $\Theta_{4}={\left(\delta \rho \ell a_{2}\right)}^{-1}$.

Based on [62], Equation (7.811.5), and applying some polynomial expansion manipulations, $C_{D_{1}}^{ipSIC}$ is given by

(50) $$C_{D_{1}}^{ipSIC}=\frac{1}{\ln2\Gamma (\xi_{1})}\left[G_{2,3}^{3,1}\left(\zeta_{1}\Theta_{3}\left|\begin{array}{c} 0,1\\ 0,\xi_{1},0 \end{array}\right.\right)-G_{2,3}^{3,1}\left(\zeta_{1}\Theta_{4}\left|\begin{array}{c} 0,1\\ 0,\xi_{1},0 \end{array}\right.\right)\right].$$

### 5.3. Ergodic Rate of $D_{1}$ with Perfect SIC

The ergodic rate of device $D_{1}$ with ℓ=0 for the pSIC case is similar to that of the ipSIC case:

(51) $$\begin{array}{cc} C_{D_{1}}^{pSIC}= & E\left\{\log_{2}\left(1+\gamma_{D_{1}}\right)\right\}\\ = & E\left\{\log_{2}\left(1+a_{1}\delta \rho A_{1}\right)\right\}\\ = & \frac{1}{\ln2}\int_{0}^{\infty }\frac{1}{1+x}\left[1-F_{A_{1}}\left(x\right)\right]dx. \end{array}$$

Plugging (27b) into (51), we have

(52) $$C_{D_{1}}^{pSIC}=\frac{1}{\ln2\Gamma (\xi_{1})}\int_{0}^{\infty }\frac{1}{1+x}G_{1,2}^{2,0}\left(\frac{\zeta_{1}x}{\delta \rho a_{1}}\left|\begin{array}{c} 1\\ \xi_{1},0 \end{array}\right.\right)dx.$$

With the help of [62], Equation (7.811.5), $C_{D_{1}}^{pSIC}$ can be further computed by

(53) $$C_{D_{1}}^{pSIC}=\frac{1}{\ln2\Gamma (\xi_{1})}G_{2,3}^{3,1}\left(\frac{\zeta_{1}}{\delta \rho a_{1}}\left|\begin{array}{c} 0,1\\ 0,\xi_{1},0 \end{array}\right.\right).$$

### 5.4. Average Symbol Error Rate

Let us denote *p* and *q* as constants. In particular, the modulation types depend on the values of *p* and *q*. We treat the binary phase-shift keying (BPSK) modulation corresponding to p=1, q=2. If the values are p=2, q=1, they represent quadrature phase-shift keying (QPSK) and 4-quadrature amplitude modulation (4-QAM), as shown in [65], where $Q\left(.\right)$ is the Gaussian error function. For the RIS-aided point-to-point system, the average symbol error rate (SER) needs to be computed as [65]

(54) Sj=pEQqγj=p2π∫0∞Fγjx2qe−x22dx=y=Δx2x2qqpq22π∫0∞e−q2yyFγjydy,j∈D1,D2

### 5.5. Average SER of $D_{2}$

From (32), the average SER of $D_{2}$ is calculated as

(55) $$\begin{array}{cc} S_{D_{2}}= & \frac{p\sqrt{q}}{2\sqrt{2\pi }}\int_{0}^{\infty }\frac{e^{-\frac{q}{2}x}}{\sqrt{x}}F_{\gamma_{D_{2}}}\left(x\right)dx\\ = & \frac{p\sqrt{q}}{2\sqrt{2\pi }}\left[\int_{0}^{\infty }\frac{e^{-\frac{q}{2}x}}{\sqrt{x}}dx-\frac{1}{\Gamma (\xi_{2})}\int_{0}^{\frac{a_{2}}{a_{1}}}\frac{e^{-\frac{q}{2}x}}{\sqrt{x}}\Gamma \left(\xi_{2},\zeta_{2}\frac{x}{\delta \rho \left(a_{2}-xa_{1}\right)}\right)dx\right]. \end{array}$$

For the first integral in (55), using [62], Equation (3.361.2), we have

(56) $$\int_{0}^{\infty }\frac{e^{-\frac{q}{2}x}}{\sqrt{x}}dx=\sqrt{\frac{2\pi }{q}}.$$

For the second integral, let $t=\frac{2a_{1}x}{a_{2}}-1\to \frac{a_{2}\left(t+1\right)}{2a_{1}}=x\to \frac{a_{2}}{2a_{1}}dt=dx$, and we have

(57) $$\begin{array}{cc} \; & \frac{1}{\Gamma (\xi_{2})}\int_{0}^{\frac{a_{2}}{a_{1}}}\frac{e^{-\frac{q}{2}x}}{\sqrt{x}}\Gamma \left(\xi_{2},\zeta_{2}\frac{x}{\delta \rho \left(a_{2}-xa_{1}\right)}\right)dx\\ \; & =\frac{a_{2}}{2a_{1}\Gamma \left(\xi_{2}\right)}\int_{-1}^{1}\frac{e^{-\frac{q}{2}\Lambda \left(t\right)}}{\sqrt{\Lambda \left(t\right)}}\Gamma \left(\xi_{2},\zeta_{2}\frac{\Lambda \left(t\right)}{\delta \rho \left(a_{2}-\Lambda \left(t\right)a_{1}\right)}\right)dt, \end{array}$$

where Λt=a2t+1a2t+12a12a1. Unfortunately, finding a closed-form expression for the second integral is a challenging task, but an accurate approximation can be obtained for it. By using the Gaussian–Chebyshev quadrature ([66], Equation (25.4.38)), it can be achieved by

(58) $$\begin{array}{cc} \; & \frac{a_{2}}{2a_{1}\Gamma \left(\xi_{2}\right)}\int_{-1}^{1}\frac{e^{-\frac{q}{2}\Lambda \left(t\right)}}{\sqrt{\Lambda \left(t\right)}}\Gamma \left(\xi_{2},\zeta_{2}\frac{\Lambda \left(t\right)}{\delta \rho \left(a_{2}-\Lambda \left(t\right)a_{1}\right)}\right)dt\\ \; & \approx \frac{\pi a_{2}}{2Wa_{1}\Gamma \left(\xi_{2}\right)}\sum_{w=1}^{W}\sqrt{1-\chi_{w}^{2}}\frac{e^{-\frac{q}{2}\Lambda \left(\chi_{w}\right)}}{\sqrt{\Lambda \left(\chi_{w}\right)}}\Gamma \left(\xi_{2},\zeta_{2}\frac{\Lambda \left(\chi_{w}\right)}{\delta \rho \left(a_{2}-\Lambda \left(\chi_{w}\right)a_{1}\right)}\right), \end{array}$$

where $\chi_{w}=\cos\left(\frac{2w-1}{2W}\pi \right)$ and *W* is a complexity–accuracy tradeoff parameter.

Now, substituting (58) and (56) into (55), the average SER of $D_{2}$ is given by

(59) $$S_{D_{2}}\approx \left[\frac{p}{2}-\frac{p\sqrt{q\pi }a_{2}}{2^{2.5}Wa_{1}\Gamma \left(\xi_{2}\right)}\sum_{w=1}^{W}\sqrt{1-\chi_{w}^{2}}\frac{e^{-\frac{q}{2}\Lambda \left(\chi_{w}\right)}}{\sqrt{\Lambda \left(\chi_{w}\right)}}\Gamma \left(\xi_{2},\zeta_{2}\frac{\Lambda \left(\chi_{w}\right)}{\delta \rho \left(a_{2}-\Lambda \left(\chi_{w}\right)a_{1}\right)}\right)\right].$$

### 5.6. Average SER of $D_{1}$

The average SER in the ipSIC case for $D_{1}$ can be computed as follows:

(60) $$\begin{array}{cc} S_{D_{1}}^{pSIC}= & \frac{p\sqrt{q}}{2\sqrt{2\pi }}\int_{0}^{\frac{a_{1}}{\ell a_{2}}}\frac{e^{-\frac{q}{2}x}}{\sqrt{x}}F_{A_{1}}\left(\frac{x}{\delta \rho \left(a_{1}-x\ell a_{2}\right)}\right)dx\\ = & \frac{p\sqrt{q}}{2\sqrt{2\pi }}\left[\int_{0}^{\infty }\frac{e^{-\frac{q}{2}x}}{\sqrt{x}}dx-\frac{1}{\Gamma (\xi_{1})}\int_{0}^{\frac{a_{2}}{a_{1}}}\frac{e^{-\frac{q}{2}x}}{\sqrt{x}}\Gamma \left(\xi_{1},\frac{\zeta_{1}x}{\delta \rho \left(a_{1}-x\ell a_{2}\right)}\right)dx\right]. \end{array}$$

Similarly, by solving $S_{D_{2}}$, $S_{D_{1}}^{ipSIC}$ can be obtained as

(61) $$S_{D_{1}}^{pSIC}\approx \left[\frac{p}{2}-\frac{p\sqrt{q\pi }a_{1}}{2^{2.5}W\ell a_{2}\Gamma \left(\xi_{1}\right)}\sum_{w=1}^{W}\sqrt{1-\chi_{w}^{2}}\frac{e^{-\frac{q}{2}\Psi \left(\chi_{w}\right)}}{\sqrt{\Psi \left(\chi_{w}\right)}}\Gamma \left(\xi_{2},\zeta_{2}\frac{\Psi \left(\chi_{w}\right)}{\delta \rho \left(a_{1}-\Psi \left(\chi_{w}\right)\ell a_{2}\right)}\right)\right].$$

where Ψt=a1t+1a1t+12ℓa22ℓa2.

Next, the average SER in the pSIC case for $D_{1}$ is given by

(62) $$\begin{array}{cc} S_{D_{1}}^{pSIC}= & \frac{p\sqrt{q}}{2\sqrt{2\pi }}\int_{0}^{\infty }\frac{e^{-\frac{q}{2}x}}{\sqrt{x}}F_{\gamma_{D_{1}}}\left(x\right)dx\\ = & \frac{p\sqrt{q}}{2\sqrt{2\pi }}\int_{0}^{\infty }\frac{e^{-\frac{q}{2}x}}{\sqrt{x}}\left[1-\frac{1}{\Gamma \left(\xi_{1}\right)}\Gamma \left(\xi_{1},\frac{\zeta_{1}x}{\delta \rho a_{1}}\right)\right]dx\\ = & \frac{p\sqrt{q}}{2\sqrt{2\pi }}\left[\int_{0}^{\infty }\frac{e^{-\frac{q}{2}x}}{\sqrt{x}}dx-\frac{1}{\Gamma \left(\xi_{1}\right)}\int_{0}^{\infty }\frac{e^{-\frac{q}{2}x}}{\sqrt{x}}G_{1,2}^{2,0}\left(\frac{\zeta_{1}x}{\delta \rho a_{1}}\left|\begin{array}{c} 1\\ \xi_{1},0 \end{array}\right.\right)dx\right]. \end{array}$$

For the first integral, it is solved in formula as follows: (56) $\int_{0}^{\infty }\frac{e^{-\frac{q}{2}x}}{\sqrt{x}}dx=\sqrt{\frac{2\pi }{q}}$. For the second integral, using similar methods as in [62,67], (7.813.1), we have

(63) $$\frac{1}{\Gamma \left(\xi_{1}\right)}\int_{0}^{\infty }\frac{e^{-\frac{q}{2}x}}{\sqrt{x}}G_{1,2}^{2,0}\left(\frac{\zeta_{1}x}{\delta \rho a_{1}}\left|\begin{array}{c} 1\\ \xi_{1},0 \end{array}\right.\right)dx=\frac{1}{\Gamma \left(\xi_{1}\right)}{\left(\frac{2}{q}\right)}^{\frac{1}{2}}G_{2,2}^{2,1}\left(\frac{2\zeta_{1}}{\delta q\rho a_{1}}\left|\begin{array}{c} 0.5,1\\ \xi_{1},0 \end{array}\right.\right).$$

Substituting (63) and (56) into (62) and performing some mathematical manipulations, we obtain the ASER of $D_{1}$ with pSIC as

(64) $$S_{D_{1}}^{pSIC}=\left[\frac{p}{2}-\frac{p}{2\sqrt{\pi }\Gamma \left(\xi_{1}\right)}G_{2,2}^{2,1}\left(\frac{2\zeta_{1}}{\delta q\rho a_{1}}\left|\begin{array}{c} 0.5,1\\ \xi_{1},0 \end{array}\right.\right)\right].$$

## 6. Optimal Performance Analysis

We can see from (37), (36) and (34) that the OP is related to the time-switching (TS) factor $\alpha_{i}$. As a result, in order to minimize the likelihood of an outage, we must discover the best solution of $\alpha_{i}$, which can be expressed as

(65) $$\begin{array}{cc} \alpha_{i}^{*}= & \arg\min_{\alpha_{i}}P_{D_{i}}\\ \; & s.t.\;\;0<\alpha_{i}<1 \end{array}$$

where $i\in \left\{1,2\right\}$ and $\alpha_{i}^{*}$ denotes the optimal solution of $\alpha_{i}$ for minimizing the system’s OP.

We know from (35) and (33) that $R_{i}$ is a fixed number and that there is a positive correlation between the OP and $\gamma_{{th}_{i}}$. This suggests that the solution to (65) is unique and can be solved using a linear search strategy, such as the golden section method. So, we use the golden section method to solve the optimization problem, and the specific process is presented in Algorithm 1.

 **Algorithm 1: **Optimization algorithm to find $\alpha_{i}^{*}$ based on the golden section method.  **Input:** Initialize $\psi_{\min}^{i}=0$, $\psi_{\max}^{i}=1$, the golden section search $\varpi^{i}=\frac{\sqrt{5}-1}{2}$ and a      stopping threshold $\Delta^{i}=10^{-3}$  **Output:** The optimal of $\alpha_{i}^{*}$ that minimum the OP $P_{D_{i}}\left(\alpha_{i}^{*}\right)$  
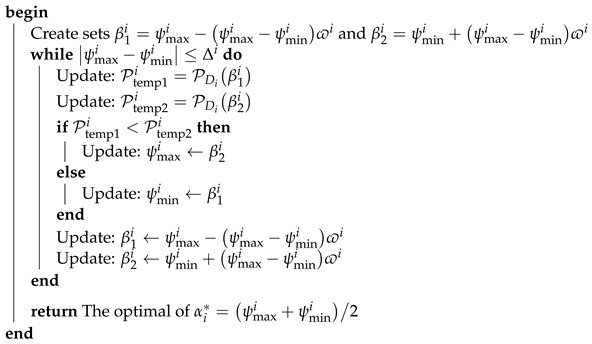


## 7. Numerical Results

In this section, we quantitatively assess our theoretical conclusions regarding the OP and ergodic rate performance. We define the fading parameters as $m=m_{h}=m_{g_{0}}=m_{g_{1}}=m_{g_{2}}$. Monte Carlo simulation [68,69,70,71,72] results are averaged across $10^{6}$ independent trials. The target rate is represented in BPCU (bits per channel use). In the figures below, we represent “Ana.”, “Sim.”, and “Asymp.” as analytical analysis, simulation, and asymptotic computation, respectively. Table 2 summarizes the other major parameters. The parameters of the κ−μ random variables are presumed to be $k=k_{h}=k_{g_{0}}=k_{g_{1}}=k_{g_{2}}$ and $\mu =\mu_{h}=\mu_{g_{0}}=\mu_{g_{1}}=\mu_{g_{2}}$ (see Table 3 for examples). In addition, the Gauss–Chebyshev parameter is chosen as W=100 to obtain a near approximation.

**Figure 2:**
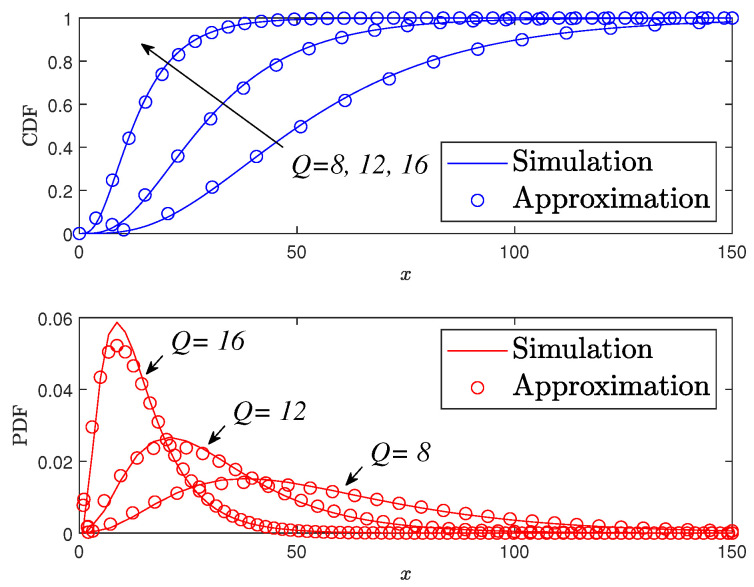
PDF and CDF for different numbers of STAR-RIS elements (Q=8,12,16), with κ=1 and μ=4.

Figure 2 illustrates the probability density function (PDF) and cumulative distribution function (CDF) for different numbers of STAR-RIS elements (*Q* = 8, 12, 16). The top graph shows the CDF, where the curves shift to the right as the number of elements increases, indicating a higher probability of achieving larger values of the random variable *x*. The bottom graph shows the PDF, where the curves become narrower and taller as the number of elements increases, suggesting a more concentrated distribution around the mean value of *x*. The simulation results, represented by circles, closely match the approximation results, represented by lines, validating the approximation method’s accuracy.

**Figure 3:**
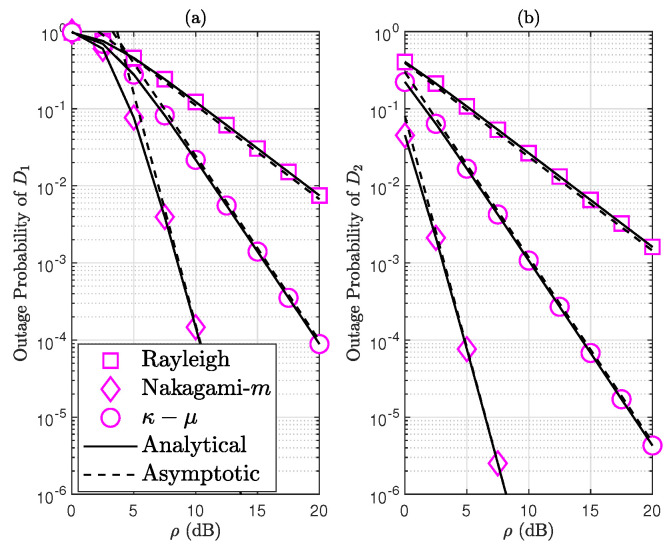
Comparison of OPs in a classic channel with varying numbers of STAR-RIS elements Q=16.

Figure 3 demonstrates the OPs of users $D_{1}$ (in subfigure a) and $D_{2}$ (in subfigure b) in a STAR-RIS-assisted NOMA system under various classical channel models (Rayleigh, Nakagami-m, and Rician) and transmit power levels ρ. The OP is plotted on a logarithmic scale against the transmit power in dB. The results show that the OP decreases as the transmit power increases for all channel models. Additionally, the Rician channel model consistently exhibits the lowest OP, followed by Nakagami-m and Rayleigh. The analytical results closely match the asymptotic results, validating the accuracy of the analysis.

**Figure 4:**
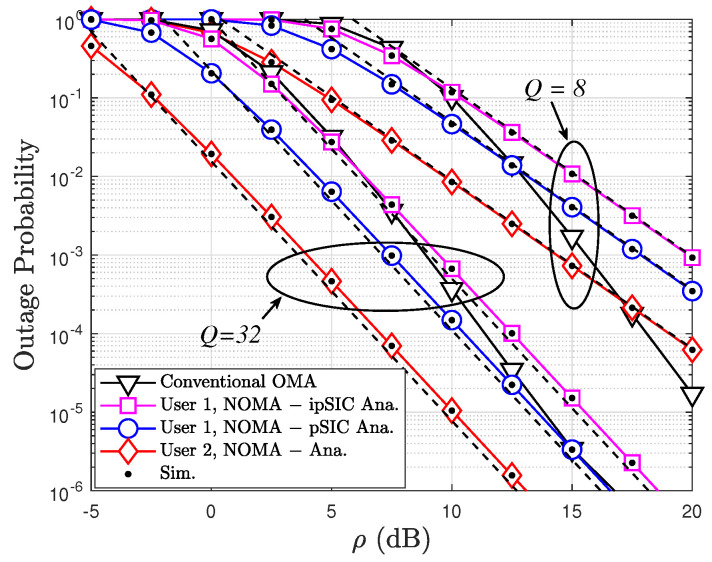
OPs of two users versus ρ with varying numbers of STAR-RIS elements Q=8,32, κ=0, and μ=2.

Figure 4 provides a comparative analysis of the OPs of two users (User 1 and User 2) in both STAR-RIS-assisted NOMA and conventional OMA systems. The OP is depicted as a function of the transmit power (ρ) in dB, with varying numbers of STAR-RIS elements (*Q*) ranging between 4 and 16. The results reveal several key insights. First, the OP for both NOMA and OMA users decreases as the transmit power increases, highlighting the positive impact of higher power levels on signal quality and reception. Second, the incorporation of STAR-RIS elements significantly enhances performance for both NOMA and OMA users. This improvement can be attributed to the superior signal focusing and interference mitigation capabilities of STAR-RIS, which become more pronounced with an increasing number of elements. Furthermore, the STAR-RIS-assisted NOMA system consistently outperforms the conventional OMA system in terms of the OP, particularly for User 2. This advantage stems from NOMA’s ability to serve multiple users simultaneously on the same time-frequency resource, leading to improved spectral efficiency. However, within the NOMA scenario, User 1 experiences a higher OP compared to User 2 due to the successive interference cancellation (SIC) process. In this process, User 1 decodes its own signal while treating User 2’s signal as interference, leading to residual interference and a higher OP. In addition, User 1 experiences different outage probabilities under imperfect successive interference cancellation (ipSIC) and perfect successive interference cancellation (pSIC). With ipSIC, residual interference from User 2’s signal degrades User 1’s signal quality, leading to a higher OP. Conversely, with pSIC, User 1 perfectly cancels this interference, resulting in a lower OP. This difference highlights the importance of effective SIC techniques in NOMA systems. The close alignment between the simulation and analytical results validates the accuracy of the analytical model employed to derive the OP expressions.

**Figure 5:**
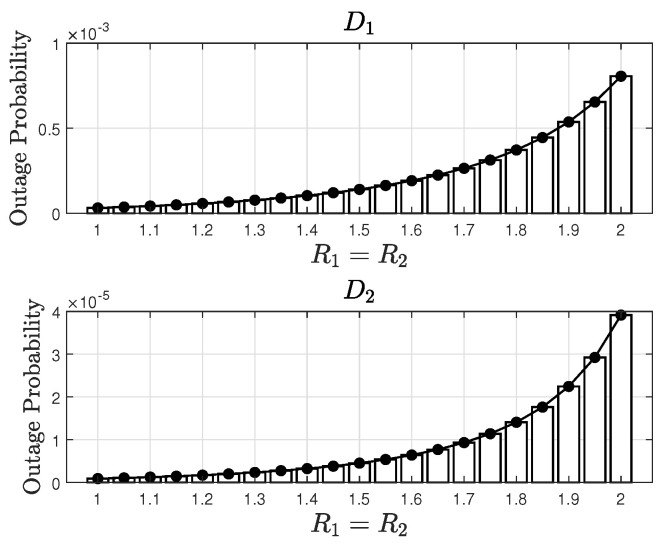
OP versus the target rates with Q=16, $a_{2}=0.9$, $a_{1}=0.1$, κ=2, μ=0, ℓ=0.01, and ρ=11 dB.

Figure 5 illustrates the OPs of users $D_{1}$ (top subfigure) and $D_{2}$ (bottom subfigure) in a STAR-RIS-assisted NOMA system versus the target rates $R_{1}$ and $R_{2}$. The OP is plotted on a logarithmic scale. The results show that the OP increases with the target rates for both users. Additionally, the OP of user $D_{1}$ is significantly higher than that of user $D_{2}$, which is due to the SIC process in NOMA. The simulation results closely match the analytical results, thereby validating the accuracy of the analysis.

**Figure 6:**
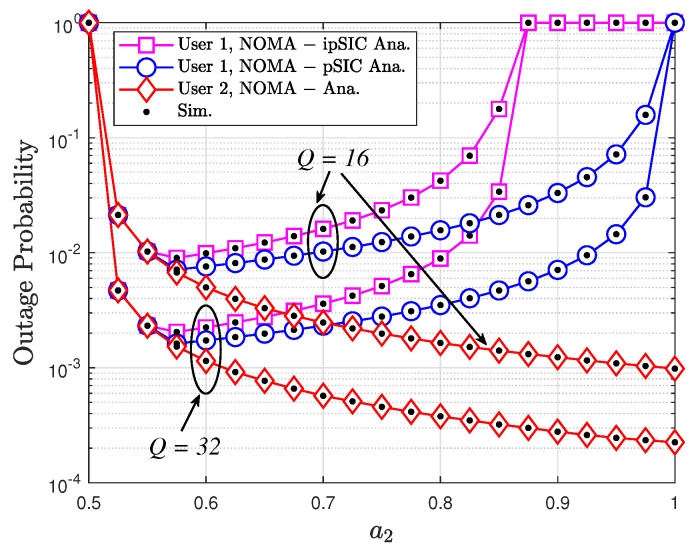
OP versus $a_{2}$ with ℓ=0.05, κ=2, μ=1, $R_{1}=1$, $R_{2}=0.5$, and ρ=5 dB.

Observing Figure 6, it is evident that the simulation and analytical results closely align, thus validating the accuracy of the analysis in illustrating the OP of users within a STAR-RIS-assisted NOMA system. The simulation specifically focuses on the impact of the power allocation factor ($a_{2}$) on the OP. It reveals that increasing $a_{2}$ initially decreases the OP for both users, but beyond a certain point, the OP of User 1 begins to rise again, while that of User 2 continues to decline. This can be attributed to the increased power allocation to User 2, which improves its performance but negatively affects User 1 due to heightened interference. The optimal value of $a_{2}$, which minimizes the OP for both users, is contingent upon specific system parameters and channel conditions. Furthermore, an increase in the number of STAR-RIS elements significantly enhances performance for both users. Notably, the OP for User 1 under ipSIC conditions is higher than that under pSIC due to residual interference, as elucidated in Figure 4.

**Figure 7:**
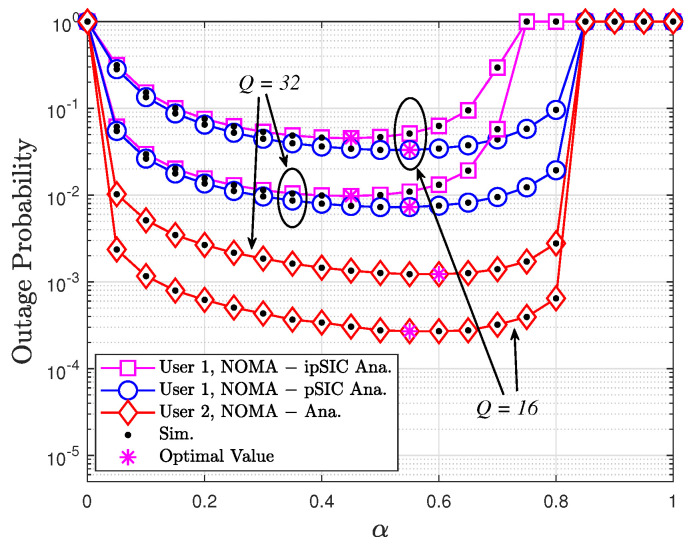
OP of the destination versus α with ℓ=0.01, κ=2, μ=1, $a_{2}=0.9$, $a_{1}=0.1$, $R_{1}=1$, $R_{2}=0.5$, and ρ=5 dB.

Figure 7 clearly shows the OPs of users in a NOMA system as a function of the fraction of block time (α), considering both perfect SIC (pSIC) and imperfect SIC (ipSIC) scenarios, with varying numbers of reflecting elements (8 or 16). The simulation reveals that increasing the number of STAR-RIS elements significantly enhances performance for both users. Notably, the OP for User 1 under ipSIC conditions is higher than the OP under pSIC due to residual interference, as illustrated in Figure 4. In addition, as α increases, the OP for both users initially decreases, reaching an optimal point, and then starts to increase. Specifically, when α = 0.4, User 1 in the ipSIC scenario achieves the best performance. Similarly, for User 1 in the pSIC scenario and User 2, the optimal α values are α = 0.5 and α = 0.6, respectively. This facilitates the determination of the optimal α value to minimize the OP for a given system configuration.

**Figure 8:**
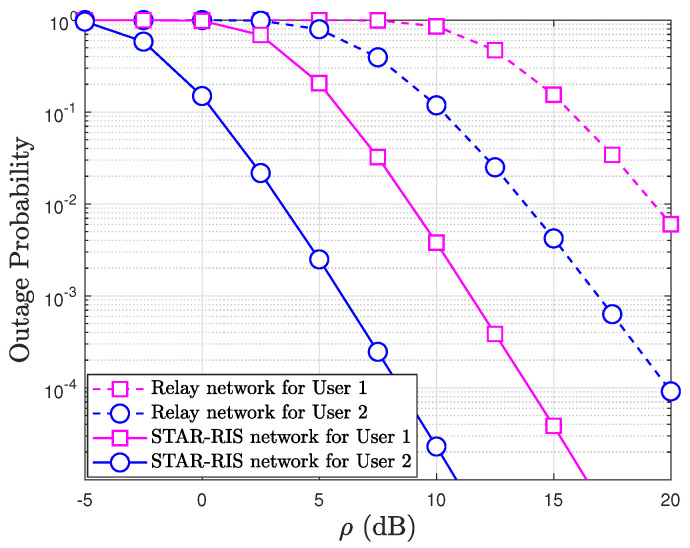
OP of the STAR-RIS-aided NOMA: Relay networks versus ρ with ℓ=0.01, κ=2, μ=0, $a_{2}=0.8$, $a_{1}=0.2$, $R_{1}=2$, $R_{2}=1$, and Q=8.

In Figure 8, the STAR-RIS network shows significantly better performance in terms of the outage probability compared to the relay network. The STAR-RIS network curves drop much faster, reaching lower outage probabilities as ρ increases. Both networks show that User 1 experiences a higher outage probability than User 2 for the same value of ρ. However, the performance gap between User 1 and User 2 is more pronounced in the relay network. At higher values of ρ (15–20 dB), the STAR-RIS network for User 2 approaches extremely low outage probabilities (below $10^{-4}$), while the relay network does not reach such low probabilities even at the highest ρ. The plot indicates that the STAR-RIS network provides a substantial improvement in reducing the outage probability compared to the relay network, particularly at higher SNRs. This is consistent for both User 1 and User 2, with User 2 benefiting more from both networks across the range of ρ. This figure demonstrates the clear advantage of the STAR-RIS network in improving reliability, especially in high-SNR conditions.

**Figure 9:**
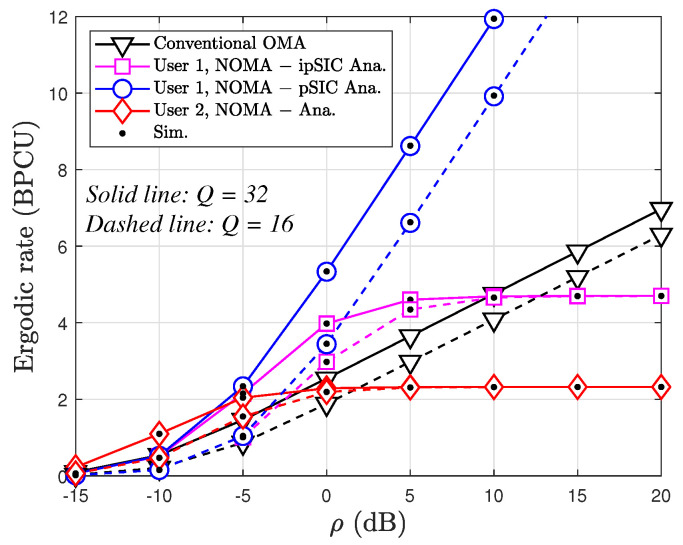
Ergodic rate versus ρ with ℓ=0.01, κ=0, μ=4, $a_{2}=0.8$, and $a_{1}=0.2$.

Figure 9 illustrates the ergodic rate (BPCU) of users in a STAR-RIS-assisted NOMA system and a conventional OMA system versus ρ under varying conditions of pSIC and ipSIC, as well as different numbers of reflecting elements (*Q* = 16 or 32). The analysis reveals several key findings. First is NOMA’s superiority. Across all scenarios, the NOMA system consistently outperforms the OMA system in terms of the ergodic rate for both users. This highlights NOMA’s inherent advantage in spectral efficiency, as it allows multiple users to share the same time-frequency resources. Second is the impact of SIC. The ergodic rate of User 1 under pSIC is significantly higher than that under ipSIC. This underscores the critical role of effective interference cancellation in NOMA. In pSIC, User 1 can perfectly decode and remove User 2’s signal before decoding its own, while ipSIC leaves residual interference, degrading User 1’s performance. This disparity widens as the transmit ρ increases, indicating that ipSIC becomes more detrimental at higher ρ levels. In addition, increasing the number of reflecting elements in the STAR-RIS from 8 to 16 substantially boosts the ergodic rate for both users in the NOMA system. This demonstrates the significant potential of the STAR-RIS to enhance the spectral efficiency of NOMA by intelligently manipulating the wireless environment.

**Figure 10:**
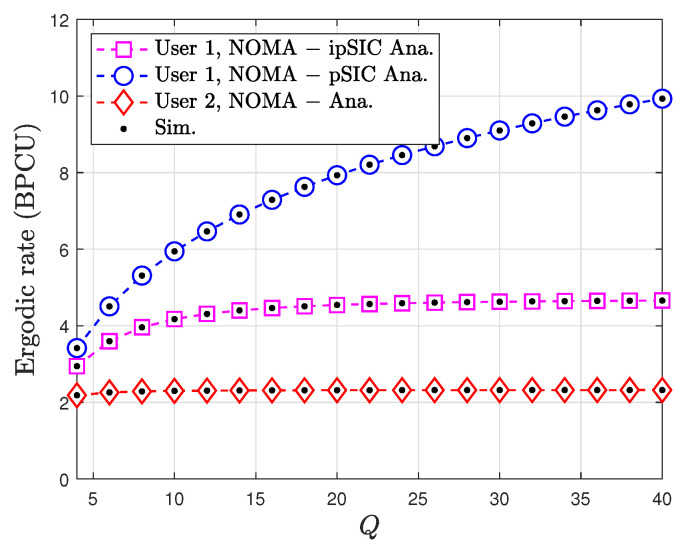
Ergodic rate versus *Q* with ℓ=0.01, κ=1, μ=3, $a_{2}=0.8$, $a_{1}=0.2$, and ρ=3 dB.

Figure 10 shows the ergodic rate, a measure of the average achievable data rate, for a two-user NOMA system as a function of the number of reflecting elements (*Q*). The number of reflecting elements is equal for both users, and the ergodic rate is plotted for both users in the NOMA system. A key observation is that the ergodic rate for both users increases as the number of reflecting elements increases. This is due to the increased signal strength and diversity gain provided by the additional signal propagation paths created by the reflecting elements. User 1 consistently achieves a higher ergodic rate than User 2 due to the power allocation strategy in NOMA, which allocates more power to the stronger user (User 1) to ensure reliable communication. The analytical results, obtained through mathematical modeling, closely match the simulation results, validating the accuracy of the analytical model in predicting the ergodic rate performance of the NOMA system. Additionally, for User 1, the ergodic rate with ipSIC is slightly lower than that with pSIC, as expected. This is because ipSIC introduces some residual interference, which degrades the performance of User 1, highlighting the importance of accurate interference cancellation in NOMA systems.

**Figure 11:**
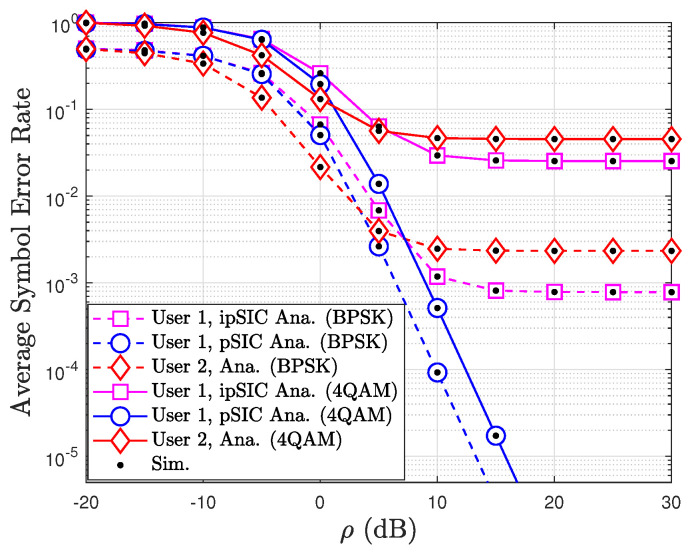
The average SERs of the considered STAR-RIS assisted NOMA system versus ρ for BPSK and 4QAM modulations with ℓ=0.05, κ=1, μ=2, $a_{2}=0.8$, $a_{1}=0.2$, and Q=8.

Figure 11 plots the average symbol error rate (SER) performance of a STAR-RIS-assisted NOMA system with two users, employing both BPSK and 4-QAM modulation schemes. The SER, a measure of the rate at which symbols are incorrectly detected, is plotted against the transmit power (ρ) in dB. The results are presented for both analytical and simulation scenarios, with and without perfect successive interference cancellation (pSIC and ipSIC, respectively). As expected, the SER for both users decreases as the transmit power increases due to the stronger received signal resulting from higher power. The 4-QAM modulation scheme, which carries more bits per symbol than BPSK, exhibits a higher SER for the same transmit power, indicating its greater susceptibility to errors in the presence of noise and interference. A significant observation is the impact of perfect SIC on User 1’s SER. The SER is notably lower when perfect SIC is employed compared to the case with imperfect SIC, highlighting the importance of accurate interference cancellation in NOMA systems for the effective removal of interference from User 2’s signal. The close alignment between the analytical and simulation results validates the accuracy of the analytical model in predicting the SER performance of the STAR-RIS-assisted NOMA system. Overall, the figure provides valuable insights into the influence of transmit power, modulation scheme, and SIC on the average SER, emphasizing the importance of optimizing these parameters to achieve the desired SER performance in practical NOMA deployments.

## 8. Conclusions

In this paper, we have investigated the performance of a STAR-RIS-assisted NOMA system with PB energy harvesting. We derived closed-form expressions for the OP and ergodic rate of the system, considering both perfect and imperfect SIC scenarios. Our analysis revealed the impact of imperfect SIC on system performance and provided insights into the design and optimization of EH–PB–STAR-RIS–NOMA systems. We also presented extensive numerical results to validate the accuracy of our analytical model and demonstrate the superiority of the proposed framework compared to conventional NOMA and OMA systems. Our results show that the proposed framework can significantly improve the OP and ergodic rate of NOMA systems, especially in the presence of blockages. To achieve the best performance for the STAR-RIS-assisted NOMA system, it is crucial to determine the optimal values for various system parameters. These parameters include the power allocation coefficients ($a_{1}$ and $a_{2}$), the time-switching factor (α), and the number of reflecting elements (*Q*). The optimal values can be determined by analyzing the derived closed-form expressions for the OP and ergodic rate and by conducting simulations under different scenarios. For instance, Figure 6 illustrates the impact of the power allocation factor ($a_{2}$) on the OPs of both users. By finding the value of $a_{2}$ that minimizes the OP for both users, we can determine the optimal power allocation strategy. Similarly, Figure 7 shows the effect of the time-switching factor (α) on the OP. The optimal α value can be determined by identifying the point where the OP is minimized for both users. By carefully optimizing these parameters, we can maximize the performance of the STAR-RIS-assisted NOMA system in terms of the OP and ergodic rate while also ensuring its practicality and cost-effectiveness. In conclusion, the proposed EH–PB–STAR-RIS–NOMA framework offers a promising solution to overcome the challenges of blockages and limited energy in wireless communication systems. By integrating STAR-RIS with NOMA and PB energy harvesting, we can achieve self-sustainable, energy-efficient, and high-performance wireless networks. The analytical and simulation results presented in this paper provide valuable insights for the design and optimization of such networks.

## Figures and Tables

**Table 2:** Main parameters for our simulations.

Parameter	Notation	Values
Total reflecting elements	*Q*	32
Power allocation factors	$\left\{a_{1},a_{2}\right\}$	$\left\{0.2,0.8\right\}$
Target rates used to decode $x_{1}$ and $x_{2}$	$\left\{R_{1},R_{2}\right\}$	$\left\{2,1\right\}$ BPCU
The fading parameter	*m*	2
The energy conversion efficiency	η	1
The fraction of the block time	α	0.5
The efficiency of SIC for $x_{2}$	*ℓ*	0.01

**Table 3:** Common fading distributions derived from the κ−μ distribution [73].

Channel	Parameters of the κ−μ Distribution
One-sided Gaussian	$\underline{\mu }=0.5,\;\underline{\kappa }\to 0$
Rayleigh	$\underline{\mu }=1,\;\underline{\kappa }\to 0$
Nakagami-*m*	$\underline{\mu }=m,\;\underline{\kappa }\to 0$
Rician with parameter *K*	$\underline{\mu }=1,\;\underline{\kappa }=K$
κ−μ	$\underline{\mu }=\mu ,\;\underline{\kappa }=\kappa$

## Data Availability

The original contributions presented in the study are included in the article, further inquiries can be directed to the corresponding author.
